# Supplementary material for: A Systematic Review on COVID-19 Vaccine Strategies, Their Effectiveness, and Issues
Source: Vaccines (Basel). 2021 Nov 24;9(12):1387. doi: 10.3390/vaccines9121387 (PMC8708628; doi:10.3390/vaccines9121387)
Supplement: Supplementary file 1 [file vaccines-09-01387-s001.zip › vaccines-1421083-supplementary.pdf]

Table S1. Search strategy.

Database	Search Strategy
**PubMed**	Advanced search: ((covid-19[Title/Abstract]) OR (sars-cov-2[Title/Abstract]) OR (novel coronavirus[Title/Abstract]) OR (n-CoV[Title/Abstract])) AND (vaccine[Title/Abstract]))<br>&<br>((covid-19[Title/Abstract]) OR (sars-cov-2[Title/Abstract]) OR (novel coronavirus[Title/Abstract]) OR (n-CoV[Title/Abstract])) AND (vaccine[Title/Abstract]) AND (efficacy[Title/Abstract]))<br>&<br>((covid-19[Title/Abstract]) OR (sars-cov-2[Title/Abstract]) OR (novel coronavirus[Title/Abstract]) OR (n-CoV[Title/Abstract])) AND (vaccine[Title/Abstract]) AND (seroconversion[Title/Abstract]))<br>&<br>((covid-19[Title/Abstract]) OR (sars-cov-2[Title/Abstract]) OR (novel coronavirus[Title/Abstract]) OR (n-CoV[Title/Abstract])) AND (vaccine[Title/Abstract]) AND (side effect[Title/Abstract]))
**ScienceDirect**	Advanced search Keywords: “Covid-19”/“SARS-CoV-2”/“novel coronavirus”/“n-CoV” “vaccine” “RCT”/“Randomized controlled trial”/“Clinical trial”/“clinical study” “efficacy” “seroconversion” “side effect”
**Google Scholar**	allintitle: (“Covid-19” OR “SARS-CoV-2” OR “novel coronavirus” OR “n-CoV”) “vaccine” (“RCT” OR “Randomized controlled trial” OR “Clinical trial” OR “clinical study”) “efficacy” “seroconversion” “side effect”
